# Taxing reproduction: the full transfer cost of rearing children in Europe

**DOI:** 10.1098/rsos.230759

**Published:** 2023-10-11

**Authors:** Pieter Vanhuysse, Márton Medgyesi, Róbert Iván Gál

**Affiliations:** ^1^ Full Professor of Politics and Public Policy, Department of Political Science and Public Management; Senior Fellow of Business and Social Sciences, Danish Institute for Advanced Study; Member of the European Academy, University of Southern Denmark, Campusvej 55, 5230 Odense M, Denmark; ^2^ Senior Researcher, TARKI Social Research Institute; Senior Researcher, Child Opportunities Research Group, HUN-REN Centre for Social Sciences; Senior Researcher, Corvinus Institute for Advanced Studies, Corvinus University, Budapest, Hungary; ^3^ Head of Research Centre, Corvinus Institute for Advanced Studies; Associate Professor, Department of Sociology, Corvinus University, Budapest, Hungary; ^4^ Senior Researcher, Hungarian Demographic Research Institute; Visiting Professor, Institute of Economics, Hitotsubashi University, Kunitachi, Tokyo, Japan

**Keywords:** national transfer accounts, valuing care, societal reproduction, unpaid household labour, intergenerational transfers, social policy

## Abstract

What are the intergenerational resource transfer contributions of parents and non-parents in Europe? Using National Transfer Accounts and National Time Transfer Accounts for 12 countries around 2010, we go beyond public transfers (net taxes) to also value two statistically much less visible transfers in the family realm: of market goods and of unpaid household labour (time). Non-parents contribute almost exclusively to public transfers. But parents additionally provide still larger private transfers: mothers mainly time, fathers mainly market goods. Estimating transfer stocks over the working life, the average parental/non-parental contribution ratio in Europe flips from 0.73 (public transfers alone) to 2.66 (all three transfers combined). The highest combined parental/non-parental contribution ratios are in Sweden and Finland. The metaphorical tax rates implicitly imposed thereby on rearing children in Europe are multiples of the value-added tax rates in place on consumption goods. Unveiling the sheer magnitude of these invisible transfer asymmetries carries multiple implications for policy debates. For instance, it raises the question whether ageing European societies unwittingly tax, rather than subsidise, their own reproduction. Family friendly policy models, such as the Nordic welfare states, do not mitigate this effect. They help parents work, but do not lower the implicit tax parents pay.

## 1. Introduction: shining a light also on the less visible resources parents contribute

People in all post-hunter–gatherer societies face a fundamental life cycle consumption financing problem: productivity is concentrated in mid-life, but consumption is spread more uniformly over the life cycle [1]. In early and late life, we therefore generally consume more resources than we produce, while the opposite holds true in mid-life. There are few individual or market solutions to this life cycle consumption financing problem. Children and youth, in addition to facing competence limitations in dealing with their needs themselves, face major credit constraints [2]. In turn, stocking consumption goods for late life is not possible even for adults. Commodities are, by and large, perishable, services cannot be stored and tastes change over time [3]. In other words, there are strong limits to any intertemporal reallocations between one single person over his/her life cycle. Instead, early-life and late-life consumption financing require solutions that are cross-sectional and exploit the fact that generations overlap. At any time, different birth cohorts live together as different age groups. Hence ‘net resource-productive’ mid-life age groups can contribute resources to finance the consumption of those who are resource dependent in early-life and late-life [4–7].

This intergenerational solution to the life cycle consumption financing problem can be accomplished through various combinations of three channels: (i) private time transfers (redistribution of goods and services produced or provided directly by the people involved) within and between households, (ii) private ‘money' transfers (redistribution of market goods and services paid for but not directly produced or provided by the people involved) within or between households, and (iii) public transfers through net taxes and social security contributions. To illustrate with early-life resource dependency (for simplicity, ‘childhood'), this means that the responsible child-rearer (for simplicity, ‘parent') can use a combination of: (i) staying at home to care for the child her/himself (unpaid household labour), or doing paid work and either (ii) using the extra market income to buy goods and services for the child, or (iii) paying extra net taxes that can finance public child care and schooling. The need for transfers to children remains across these three channels. Switching between channels does not eliminate the parent's transfer responsibility. Instead, it largely implies a transfer conversion (on which more in §4.2): from (i) private time transfers (unpaid household labour) to (ii) private transfers of market goods and services to (iii) public transfers (net taxes).

But crucially, these three transfer types are not equally statistically visible, as they do not generate data in the same way. Familial transfers of market goods and unpaid household labour, such as the food, clothes, and care given to children, leave few traces including in statistics, as families do not usually keep accounts of such transactions. By contrast, public transfers (taxes and social security contributions) connect large groups of people and are therefore much more fully recorded and visible in national statistics. Despite key early pleas [8,9] and contemporary feminist critiques (e.g. [10–13]), this asymmetric visibility issue has been largely ignored by mainstream economics (which still predominantly focuses on market exchanges and social externalities) and mainstream social policy analysis (which predominantly focuses on state interventions).

Yet visibility matters a great deal. Since societies tend to value mainly what they measure, what is imperfectly measured tends to be both imperfectly understood and undervalued. This often has major implications, whether this regards ‘invisible women’ [14] and their ‘invisible work' [15], the crowding out of intrinsically motivated behaviour by extrinsic rewards [16], large hidden tax expenditures in seemingly lean welfare states [17], or the large hidden and often irreparable environmental costs of GDP-measured economic growth [18,19]. In this article, we argue that the higher statistical visibility of public transfers compared to private transfers of unpiaid household labour and of market goods and services similarly has major implications for how we may (mis)understand and (under)value the transfer contributions of parents in reproducing society. Our main aim is empirical: to shine a wider light on the measurement of intergenerational resource transfers by parents relative to non-parents by valuing not just public transfers but also these two types of less visible transfers in the family realm: of market goods and unpaid household labour.

The ‘green moment' in thinking about macro-economic growth came when more inclusive ways of accounting laid bare the degree to which economic production tapped into hitherto unpriced but depletable natural assets and ecological services. In the same vein, shining a wider light on less visible transfers in the family realm can show to what degree the intergenerational welfare state is surrounded by a hitherto undervalued world of transfers within households. As we show below, better accounting truly shifts perspectives in this regard, and not just marginally. The relative invisibility of parental transfers is what allows welfare states to implicitly freeride on the cost of producing their own future taxbase. To the degree that current policies and accounting procedures do not fully take into account how the next generation of taxpayers was produced in the first place, they adhere to an erroneous ‘stork theory' of child-rearing. This may lead contemporary societies, even famously family friendly welfare societies, to implicitly tax rather than subsidize their own reproduction.

To a significant degree, transfers to children also represent investments in their human capital. However, here we do not discuss the investment side of these transfers. Estimating the comparative rates of return of the various forms of private and public transfers is beyond the scope of this study. Higher levels of private investment by better-off parents in their offspring are also likely to lower intergenerational mobility in society, a topic we cannot address here [20,21]. Instead, we perform a descriptive accounting exercise that does not consider the behavioural responses of the actors and sidesteps theoretical concerns about whether or not the utility derived from childrearing sufficiently compensates parents for their expenses. We measure more fully the distribution of the input value of an activity with major social externalities—childrearing—between those who engage in it directly and those who do not.

For simplicity, below we do not refer to ‘child-rearers' and ‘non-childrearers' but to ‘parents' and ‘non-parents’. However, our use of the term ‘parent' does not fully correspond with the common-sense meaning (those who ever had children). Rather, it denotes parents who co-reside with minor children. Specifically, we aim to examine the ratio P/nP of resource contributions (P) by ‘parents’ (people who co-reside with their children) over resource contributions (nP) by ‘non-parents' (people who have no children or do not co-reside with them). Parenthood status is self-declared and the coding instructions of the questionnaires we use allow us to code as ‘parent' any adult who is identified by the respondent as someone's parent in the household, which allows us to capture also non-biological parenthood.

We focus on the cross-sectional and working-life transfer costs of rearing children, using a sample representing two-thirds of the European Union (EU) population in 2010 (pre-Brexit) and covering all main types of welfare regime: Continental (Belgium, Germany, France), Nordic (Finland, Sweden), Anglo-Saxon (UK), Mediterranean (Spain) and five institutionally heterogeneous east-central European countries (Bulgaria, Poland, Estonia, Latvia and Lithuania). We adopt definitions and models of the National Transfer Accounts methodology (henceforth NTA) and we extend it further. Electronic supplementary material, S1 appendix §S1.1, spells out in detail the methodological innovations of the NTA method in extending the standard System of National Accounts, and how we go one step beyond these innovations by also splitting age-based NTA accounts along a further age-variant variable, childrearing. More specifically, we split the age profiles of public transfers and familial transfers (of money and unpaid household labour) by parenthood status and use them in a flows-to-stock exercise to assess synthetic working-lifecourses of parents cohabiting with minor children and of non-parents (both non-parents living in childless households and non-parents cohabiting with minor children who are not their own) in terms of net transfer outflows in working age.

Sections 2 and 3 review the literature and indicate why measuring parental resource contributions better matters. Section 4 spells out our theory and expectations. Section 5 discusses definitions, data and the methods used to model the three types of intergenerational transfers. Section 6 presents cross-sectional age profiles of transfers split between parents and non-parents and a flows-to-stock exercise to assess synthetic lifecourses of parents and non-parents in terms of net transfer payment during their working lives. Sections 7, 8 and 9 widen the analysis by estimating the implicit ‘tax' rates on rearing children and by discussing the seeming Nordic paradox (higher child-rearing ‘tax' rates in these family friendly welfare states) and gender (motherhood and fatherhood). We conclude by discussing further implications for further research and societal and policy debates.

## 2. The costs of child rearing: literature review

Wide across middle-income and rich societies, social policies financed by taxpayers, such as public education and family policies, are used to socialize the cost of child-rearing [2,7]. But they do so only to a limited degree. On balance, it is actually *parents,* not taxpayers generally, who bear the lion's share of the cost of rearing children: in money and in time, directly and in opportunities foregone [11,22–28]. For most parents, the effort involved in rearing children is substantial. This has been demonstrated even in studies that do not estimate the value of parental time. For instance, [29] estimate that long-run child penalties for mothers in terms of lower labour market earnings five to ten years after childbirth are large across six OECD countries, at between 21 and 61% (see also [30–35]). As our approach constructs stylized lifecycles from cross-sectional estimations, it cannot directly take into account the parts of the motherhood penalty on lifetime earnings that derive from opportunity costs such as, for instance, skill loss, lost promotions and reduced bargaining power. This also means that we are likely to provide, if anything, conservative estimates of our main variable of interest: the relative cost of parenthood, as measured by the P/nP ratio.

Penne *et al*. [28] construct a needs-based indicator of the degree to which public transfers compensate parents for (just) the direct cost of children for two child ages in three parental income classes across six European cities. They find that public transfers compensate less than half the cost of children in 34 out of these 36 categories. Verbist & Van Lancker [36] estimate the average share of the direct cost of children that is compensated by public transfers for three family types in 31 European welfare states. They find that public transfers compensate less than half the cost in 81 out of these 93 categories. Telling as they are, these findings heavily underestimate the true cost of childrearing. Parents, especially single mothers, also dispose of significantly less discretionary time than non-parents [37]. As we show below, in addition to providing net public transfers, parents, and only parents, provide still larger private transfers: fathers mainly market goods and services (money), mothers mainly unpaid household labour (time).

Previous empirical research indicates the scale of the positive externalities parents provide. Lee & Miller [38] find that, at least in industrial societies with fertility rates around or below reproduction level, these externalities are positive and significant, amounting, for instance, to about $250 000 in today's currency for the USA (nearly three years of average salary). Folbre [39] shows that parents, especially mothers, pay most of the costs of raising the next generation, whereas employers and taxpayers derive significant net benefits. Illustrating the importance of wider accounting, Suh & Folbre [40] indicate a lower bound estimate of the replacement cost of nonmarket work of 44% of conventionally measured GDP for the USA in 2010. Wolf *et al*. [41], who do not take into consideration any private transfers, show that the combined net present value of taxes paid and benefits received for parents and their offspring in the USA exceeds that of non-parents by 66%. This article adds new and internationally comparative European evidence to the debate.

## 3. Why measuring more widely matters

These observations raise a positive question: is there significant asymmetry in the overall resource transfer contributions by parents relative to non-parents, possibly in statistically opaque ways? This question takes on added urgency on an ageing, longer-living continent with generally below-replacement fertility levels, particularly in societies with rates of intentional childlessness that are increasing, as in east-central Europe for post-1960s female cohorts, or already high, as in western and, even more so, southern Europe [42,43]. All else equal, if children raised one generation ago primarily by their own parents, rather than by general taxes or social security contributions, substantially finance the old-age consumption also of non-parents, the latter could be said to *de facto* free-ride on the collective effort provided by parents in reproducing society.

Such free-riding would matter because parents predominantly pay *privately* for the cost of rearing children to productive adulthood [10,25,38,39,41,44–47]. As we show below, this private cost can be conceived as an implicit ‘tax' on the activity of rearing children. Moreover, some of this private cost is socially imposed on parents by socio-legal obligations for continuity of adequate care [39,48,49]. To the extent that their children subsequently become ‘net resource-productive’ adults as taxpayers, social security contributors, and carer (and parents in their turn), parents create positive externalities that will benefit all of society. For instance, as adults, children will later finance public infrastructure and public pension, health and long-term care benefits—all of which will then also benefit current non-parents [41,45–47]. True enough, parental commitments are voluntary and not enforced. Moreover, parents typically do not have children with the explicit motivation of producing future taxpayers. However, neither the voluntary nature of childrearing nor its motivations in and by themselves imply any tax rate. Numerous other activities with positive externalities are voluntarily undertaken without any direct motivations to produce positive externalities, yet are not charged with such implicit taxes. Some forms of private savings and investments are even awarded tax credits to incentivize them. When people raise children, they also produce positive fiscal consequences. Yet, as we show below, instead of a tax credit, parents are charged with a significant extra implicit tax (which is likely to affect future parenting decisions). By contrast, in Ancient Rome, tax laws explicitly recognized childrearing as an activity fiscally equivalent to paying taxes. The poorest Roman citizens were tax-exempt but were considered to contribute in kind by rearing their offspring, *proles*. These poor citizens were called *proletarii:* literally, men who could serve the public only by fathering children.

To be sure, not all parental transfer inputs carry positive externalities. Children also provide important private benefits to their parents, and some part of the cost of raising them resembles pure consumption. Parents generally seem to view their children as fundamentally *sui generis* goods. They value time spent caring for children more than other household or leisure activities [50], allocate their time use accordingly [24,51], and react to material incentives rather weakly when it comes to time spent with children [52]. Rearing children, at least in high- and middle-income societies today, is thus best described deontologically as a strong intrinsic commitment [11]. But, barring immigration on a politically unrealistic scale, it is a commitment that in aggregate is a necessary precondition for the reproduction of society all the same [12,13,39,53]. This renewal of the fiscal basis of the welfare state depends on productivity-adjusted demographic continuity, which is a function of both the size (quantity) and the capabilities (quality) of successive generations.

This also raises a related normative question: to what extent *should* parents be compensated for childrearing? Why would a high parent/non-parent resource contributions ratio matter morally at all? After all, because of free self-selection into parenthood, we can assume that those whose wellbeing will be improved by having children will, on aggregate, have children—and vice versa [54]. On one side of the argument, authors focusing on the 'public good' component of children incline towards more extensive compensation of parents (e.g. [28,36])—or even a legal claim by parents on their children's earnings [10,55]. On the other side, the ‘parental provision’ argument holds that since parents were aware of children's likely costs and consequences yet freely decided to have them, they now have no moral claim on compensation for rearing children. In fact, parents should actually be taxed if their children produce 'public bads' [56,57]. Children certainly produce negative environmental externalities. Some therefore claim that procreation is equivalent to morally problematic excessive consumption—the view of children as ‘Hummers' [58–60].

Both sides of the debate, as it stands, derive their strong conclusions by exclusively focusing on either the positive or the negative externalities parents produce—not on both together. Yet children are indivisible and thus better conceptualized as three things simultaneously: consumption ‘private goods’ to their own parents, environmental ‘public bads’ to both their own and their parents' generations, and fiscal ‘public goods' to their parents' generation, whose members (including non-parents) will tomorrow depend on today's children's productive contributions. Behind a Rawlsian veil of ignorance, all members of society, irrespective of their personal conception of the good life, can be presumed to have a strong interest in having someone produce the next generation of taxpayers. This is why the public good component of childrearing has the deeper moral status of a ‘socially necessary' contribution that creates stronger obligations for all beneficiaries to share the cost [37,53].

While we remain *a priori* agnostic about the compensation question, our approach contains potentially far-reaching implications for addressing it. Whether, and to what degree, one believes that public policies should compensate parents, it is important to better measure the distributional impact of the status quo as this allows democratic debates about social reproduction and the costs of childrearing to be held on more complete and more explicit terms. Our main aim is to empirically lay bare the magnitudes of, and the asymmetries in, the contributions of parents relative to non-parents.

## 4. Theory and expectations: asymmetric socializability and the logic of transfer conversion

### 4.1. Asymmetric socializability: the prime responsibility of parents

The early-life consumption financing problem discussed in §1 is unique in a specific way. Of all the resource contributions that people in mid-life are responsible for, childrearing stands out as one for which responsibility is much less sharable between parents and taxpayers. Extensive socialization of early-life care appears to be constrained in a way that, for instance, the financing of public goods, long-term care, or pensions, is not. In all modern societies, there is a notable asymmetry in the socialization of early-life, respectively late-life needs [4]. Pre-adulthood children are raised predominantly by their own parents (a private family channel), whereas older people are predominantly supported as a generation by the generation of their adult children (a socialized, government channel). Even the large welfare states in (non-Anglo-Saxon) Europe engage in a division of labour to solve the life cycle financing problem: they are elderly-oriented welfare states embedded within societies composed of strongly child-oriented families [26]. The model of predominantly private childrearing has fully reasserted itself even in the rare settings designed explicitly to overturn it, such as collective childrearing communities in 1960s–1970s America [61] and the original Israeli kibbutz model of collective childrearing by multiple non-kin carers [62]. When the state has had to take over as a last resort, as in the case of institutionalized orphans, child developmental and adult outcomes are generally considered to be worse, even excluding those cases in which severe neglect occurs [63]. Yet, if accomplished early enough, transferring orphans to care by foster parents or adoptive parents can then often limit or reverse many of these adverse outcomes [64].

In other words, when it comes to rearing children, what parents contribute privately in money and in time is not just less visible than what taxpayers contribute. Due to asymmetric socializability, it is also to a lesser degree replaceable. Empirically, rather than normatively, the prime responsibility of parents for rearing children appears to be unavoidable. Throughout most of human evolutionary history, this responsibility was to a larger degree shared with ‘alloparents'—grandparents, aunts and uncles, and non-kin adults—through cooperative childrearing practices [65,66]. But in modern societies, the empirical reality of two-generational households has strongly reduced alloparenting and further increased the prime responsibility of parents. In contemporary Europe, the household composition structure is such that there are hardly any households in which intergenerational transfers *could* be given by anyone other than parents, such as cohabiting grandparents, uncles or aunts. Of the 140 million households in the 12 countries we study in 2010, one-third count only one person, and one-quarter is composed of couples without cohabiting children. In one-person households, no intra-household transfers take place. In households without cohabiting children, members do exchange market goods and time, but these are not intergenerational transfers as the participants tend to be close in age. In the age profile, such transfers cancel out. Of the remaining 43% of households, only a small fraction (5 p.p.) are either multi-person households composed of non-kin, such as co-residing friends (3 p.p.) or households composed of two or more families, such as three-generational households with co-residing grandparents (2 p.p.). Overall, these 5% of households can be forums for intergenerational transfers but not for parental transfers. Of course, these averages contain some cross-country variation. Whereas multigenerational households are practically non-existent in northwestern Europe, they are somewhat more present still in eastern Europe (8% of households in our Lithuanian sample, 11% in the Polish sample and 17% in the Bulgarian sample; see also [67]). We discuss grandparental contributions to childrearing below in §5.1.

Nearly all of the remaining 43% of households (38 p.p.) are either single parents raising children or couples raising children (we coded as couples households containing (a) married couples, (b) couples in registered partnership and (c) couples in consensual union). In practice therefore, intergenerational transfers within families in Europe are largely limited to nuclear households consisting exclusively of co-residing *parents* and children. In almost all households where intergenerational resource transfers can be given, they are given by parents rather than grandparents or other kin.

### 4.2. The general logic of transfer conversion: why parents pay more (everywhere)

Precisely because unpaid household labour and private market goods transfers have been hitherto much less accounted for, we expect that adding estimates of these two types of private transfers to estimates of public transfers will reveal significant asymmetries in statistical visibility of the overall resources package contributed by respectively parents and non-parents. Shining a wider light does not merely complete the picture; it may substantially change it. The virtual disappearance of three-generational households in European societies, combined with the unavoidable prime role of parental transfers, leads us to expect a further, hitherto hidden, regularity. When private transfers of market goods and services bought for cash and of unpaid household labour are included in addition to public transfers, the parental resource contributions over non-parental resource contributions ratio P/nP will significantly increase to exceed unity everywhere (the asymmetric visibility hypothesis). While many socio-economic and institutional factors may contribute to explaining variation in P/nP, we expect this ratio to be consistently above unity widely across different demographic, policy, gender relations and labour market constellations.

This is precisely because, in reality, it is parents alone who co-reside with minor children (the predominance of two-generational households today), and it is parents who are primarily responsible for childrearing (asymmetric socializability), one way or the other (the logic of transfer conversion). Simply put, non-parents nearly exclusively live either alone or in couples without co-resident minors. Hence they predominantly use just *one* channel (government) to make intergenerational contributions (though couples of course use the other two channels to make transfers among themselves). By contrast, since parents use all three channels to make intergenerational transfers, transfer conversion can occur only among them.

To illustrate the transfer conversion logic at the micro level, imagine a mother who stays at home to raise her children herself. She gives time transfers (unpaid household labour) directly to her children. If, alternatively, she takes up paid labour and hires home care help, her market income and her public transfer contributions will increase and her original time transfer will be transformed into a money transfer between her and her child through an intermediary: a market transaction between her and the hired carer. Thus the size of the overall transfer package may be less affected than its composition. If, in a third scenario, the mother works and sends her children to public daycare or kindergarten, her market income as well as her public transfers will again increase while her time transfers decrease. The original at-home time transfer will now be transformed into a parental public transfer to finance services, the recipient of which will be the children, not the parents. Again, the overall package may be largely unaffected, even though its composition changes significantly.

Clearly, policy models can affect the relative importance of the family, markets and the state in how the intergenerational resource transfer systems operate across countries. Public childcare, parental leave, family allowances, job-related benefits such as shorter working hours and care leave rights, and other work–family policies allow societies to modify the way parents, especially mothers, can combine work and family life (e.g. [68–71]). Since both men and women aspire today to combine careers with family lives, policies helping them to do so are almost certainly welfare-enhancing. Family-friendly policy bundles help parents (especially mothers) by reconciling work with family life and they help mothers by increasing their economic independence and bargaining power (though, paradoxically, they may simultaneously lower mothers' occupational and earnings attainments; see [72–74]). In so doing, family-friendly policy bundles may help to build new social foundations for a ‘return of the family’ in post-industrial economies [75–77].

Yet such family-friendly policy bundles need not imply substantially lower total transfer contributions by parents—even mothers—and may not be best conceptualized as net *extra* resources received by parents. The logic of transfer conversion implies that parents are likely to largely pay themselves for these welfare-enhancing policies through higher net tax and social security contributions. A parent taking up paid work and using public child care reduces his or her time transfers and pays more net taxes. On balance, extra revenues for governments may well exceed the cost of providing public child care [78]. We therefore expect P/nP to be well above unity even in the most family-friendly and gender-egalitarian welfare states, where (some) gender gaps and mother/non-mother gaps in overall resource contributions will be smaller as more mothers work and single-parent poverty is lower. More generally, while cross-country variation in institutions, demography, policy and gender norms likely determines the relative importance of the three types of transfers parents provide across Europe, such variation is less likely to drive the overall size of the full transfer package parents provide. The logic of transfer conversion thus leads us to expect that cross-country variation in the overall resource contributions package of parents (P) will be smaller than cross-country variation in the three types of transfer components (public transfers, market goods and services, and unpaid household labour).

## 5. Definitions, data and methods

### 5.1. Childrearing: definitions

As our focus is on the transfer costs of childrearing, we apply definitions that concentrate on dependence and transfers in terms of material resources: public transfers, money transfers (including commodities and services purchased in the market), and time transfers. We mainly use the European Union Statistics on Income and Living Conditions (EU-SILC) [79], harmonized European Household Budget Surveys (HBS) [80], the Harmonized European Time Use Survey (HETUS) [81] (on which more below), and for health-related data, the European Health Interview Survey (EHIS) [82]. None of these datasets contains information on the total number of children a person has. Instead, we exploit the data available on cohabiting persons.

This cohabitation-based definition of parenthood does not separate real-life parents from real-life childless people. Rather, it separates parents (biological or otherwise) who currently cohabit with their children from anyone else. Many parents in the everyday use of the term are not considered parents here. A biological parent is coded as a non-parent if he/she moved out of the house due to separation or divorce or if his/her children moved out. This makes non-parents in our calculations significantly more numerous and, as a group, more heterogeneous. Another feature of cohabitation-based parenthood is that it is age-variant in two ways. Real-life parenthood changes with age as children are born sequentially, but it usually remains unchanged after the number of children peaks. By contrast, coresidence-based parenthood is reversible, for instance, when real-life parents separate/divorce or when their children reach adulthood and move out. Just around the age when Europeans reach old age in the sense of becoming net resource-dependent again, the cell frequency of parents cohabiting with their children drops significantly in survey samples: beyond this age, there are hardly any parents left by the cohabitation-based definition. This limits the comparison of parents and non-parents to their working age. It also makes the comparison of real-life social groups, such as large families of low socio-economic status with three or more children and high-earning families with one or two children, more difficult. However, it does allow a near-complete and almost unhindered analysis of the transfer cost of childrearing because, as we show below, parental and non-parental transfers can be separated with a high degree of certainty using the cohabitation-based definition of parenthood.

As noted, we define a ‘parent' as a person who co-resides with at least one of his/her children, and a ‘child' as someone who co-resides with at least one of his/her parents. The latter definition is qualified with the following further specifications. First, grandparents living with their grown-up children and grandchildren are treated as parents (see electronic supplementary material, S2 appendix §S2.1). Second, everyone below age 13, even those not co-residing with a parent, is also considered a child. Third, if someone is both parent and child in a three-generational household, we consider him/her a parent but not a child. Finally, a non-parent is a person who is 13 years old or older and does not cohabit with any of his/her children (either because he/she has no children or because they do not co-reside with him/her).

### 5.2. Age profiles

The surveys used in this article allow a cross-sectional comparison of the transfer package of the average parent to that of the average non-parent. However, we also want to compare the accumulating transfer stocks over the life-course by parenthood status, not just the transfer flows of the reference year. For that purpose, we first need to go beyond the cross-sectional averages and draw the cross-sectional age profiles of parental and non-parental transfers.

Cross-sectional age profiles are frequently used to construct stylized lifecourses by assuming that current age-specific characteristics in higher ages will apply to the current young when they grow older. For instance, a current 20-year-old is assumed to have the same characteristics 20 years from now as a current 40-year-old, and so on. Some key indicators of social sciences are based on such stylized lifecourses, such as the total fertility rate, an indicator of assumed lifetime fertility derived from period age-specific fertilities; or the period life expectancy at birth, which would be the average length of life of the newborn cohort should they go through the current period age-specific mortality patterns over their life-course. A special case of this period-to-longitudinal methodology is applied to assess the value of stocks accumulating over time. For instance, the cross-sectional age profile of savings can be used to approximate the accumulating wealth [83,84].

The period-to-longitudinal (and, within it, flows-to-stock) methodology represents a stylized future scenario to assess what would happen if the age profiles remained unchanged. It has a solid analytical value but is not designed to accurately forecast the future. The conditions under which the cross-sectional distribution properly represents the life-course distribution are restrictive. The lifetime patterns of the currently young can significantly deviate from what today's period age profiles describe. In addition, the choice of the parameters used to calculate present values, such as the growth and discount rates, strongly affect the outcome. However, the ratio between two stocks estimated the same way is essentially stable because the effects of parameter change mostly cancel out by dividing one present value by the other. So the usual qualifications to the applicability of the flows-to-stock methodology do not perceptibly affect our calculations (we will return to this issue below).

While drawing the age profiles of transfers, we generally follow the methodological standards of National Transfer Accounts, a recent development in national accounting specifically designed to capture age-related economic and social issues in a comprehensive and consistent way [85–88].

### 5.3. Public transfers

We construct age profiles for public transfers, private money transfers and private time transfers. The first type, public transfers, includes all taxes, social contributions, and other forms of public revenues collected, and all cash benefits, in-kind services and public goods paid for by what public statistics call the general government: the central government (at both levels in countries having a federal structure), local governments, social security funds and other public funds. Based on household survey information, we distribute the aggregates by age, covering the entire population, including those who do not pay a particular form of tax or receive a particular form of benefit or service. The survey-estimated age profiles are adjusted to SNA and the corresponding NTA aggregates, ensuring that the entire public sector and the entire population are covered. Our reference year is 2010.

Tables 1 and 2 summarize the key methodological decisions about the construction of age profiles of public expenditures and taxes and contributions. The tables specify the SNA aggregates the age profiles are adjusted to, the functions of public spending, the sources of the aggregate data, the level of reporting data in the surveys (individual or household), the incidence assumptions and the sources of the microdata. Electronic supplementary material, S1 appendix §§S1.2 and S1.3, provide further details.

**Table 1.** Summary of the methodology of constructing the benefit age profiles. COFOG, classification of the functions of government. Unit of use: days in hospital, visits to the general practitioner and outpatient centre. Imputation methods are described in electronic supplementary material, S1 appendix §S13.

aggregates			age profiles		
SNA indicator	function (COFOG)	source of data	level of reporting	distribution of benefits (access and amount)	source of data
pure public goods (collective consumption expenditure of general government)		Eurostat, gov_10a_exp		uniform	EU-SILC (weighted total population)
in-kind benefits (individual consumption expenditure of general government)		Eurostat, gov_10a_exp			
	education—in kind (by level)		individual	access: attendance by educational level; amount: per capita spending by educational level	EU-SILC (individual attendance by level; total number of students by level)
	healthcare (by service category)		individual	access: belonging to risk group (defined by age, education and gender); amount: pattern of using services by risk group and service category priced by unit cost of use by service category	EHIS information imputed into EU-SILC (pattern of using services by risk group and service category); EU-SILC (total number of uses by risk group and service category)
cash benefits (social benefits other than social transfers in kind)		Eurostat, gov_10a_exp			
	education—cash		individual	access and amount reported in the dataset	EU-SILC
	old-age		individual		
	survivors		individual		
	sickness/disability		individual		
	unemployment		individual		
	family/children		household	distributed equally among adults in household	
	housing		household		
	other social protection, cash		household

**Table 2.** Summary of the methodology of constructing the age profiles of taxes/contributions. DG TAXUD: Directorate General for Taxation and Customs Union. The NTA equivalence scale assigns a weight of 0.4 for those age 4 or younger, increases linearly from age 4 to age 20, and is equal to 1 for adults age 20 and older. Imputation methods are described in electronic supplementary material, S1 appendix §S1.3.

	aggregates			age profiles		
	**SNA indicator**	**type of tax**	**source of data**	**level of reporting**	**incidence**	**source of data**
direct taxes	current taxes on income; net social contributions		Eurostat National Tax Lists	household	labour income	EU-SILC
	current taxes on wealth		Eurostat National Tax Lists	household	household head	EU-SILC
indirect taxes	taxes and subsidies on products					
		VAT	Eurostat National Tax Lists; CPB, 2013 (VAT rates)	estimated from household consumption and VAT rates	NTA equivalence scale	HBS information imputed into EU-SILC
		excise tax	Eurostat National Tax Lists; Excise Duty Tables of DG TAXUD (excise taxes)	individual/household	fuel consumption equivalence scale (fuel); individual consumption (alcohol, tobacco)	HBS and EHIS information imputed into EU-SILC
	other taxes and subsidies on production		Eurostat National Accounts		overall age profile of taxes on products	
other	other current transfers		Eurostat National Accounts		uniform	

### 5.4. Familial 'money' transfers: market goods and services

Disposable income is further redistributed within households (when, for instance, parents spend their earnings on goods and services for their dependent children) and between households (when pensioners support their adult children). This tertiary redistribution, however, is not included in standard national accounts or government statistics. As an important novelty, NTA models private *money* transfers, the redistribution of financial resources and commodities bought in the market within and between households, making it more suitable for analysing intergenerational transfers than previous data structures. Money transfers include, for instance, the food and clothes consumed by children as paid for by their parents or the utilities and other ‘household public goods' consumed by all household members, including those who do not contribute to them. Intra-household money transfers typically do not change hands as a particular act of giving and receiving; in fact, they are typically not even identified as ‘transfers' in the everyday meaning of the term. Parents who buy food for their children perceive it as a cost but would not usually call this a transfer in a questionnaire.

Such intra-household money transfers cannot be directly observed but have to be modelled. Providers of such transfers are household members whose individual resources (net income from labour and public cash transfers received) exceed the amount they consume. Beneficiaries are the other way around. Separately, both providers and beneficiaries can be identified straightforwardly. However, they cannot necessarily be assigned *together* to specific transfers (such as electricity used by a member and its bill paid by others).

Household members who have a deficit (consume more than their resources would allow) receive transfers from members who have a surplus. A set of sharing rules covering potential instances of household-level deficits/surpluses define the process and outcome of intra-household redistribution. Surplus members transfer the same share of their excess resources: the procedure sets a household-specific ‘transfer rate' (sometimes called a ‘family tax rate') specified by the rate of household-level aggregate deficits and surpluses and applied to the individual surpluses. The household head acts as one of the household members, but he/she also collects the outstanding surpluses/deficits and saves them (outstanding surpluses) or finances them from asset-based revenues or dissaving (outstanding deficits). It is also the household head who redistributes as transfers the individual shares of the imputed rent emanating from the ownership of owner-occupied houses. The age profiles of resources and uses are adjusted so that the population-weighted aggregates match the aggregates of national accounts. This way, the resulting age profile of intra-household transfers is consistent with the SNA. This guarantees that the calculation covers the entire economy and the entire population.

Not all familial transfers take place within households, but the relative importance of inter-household transfers dwarfs in comparison with intra-household transfers. Redistribution through public channels mobilizes 46% of net national income in our 12-country sample. Familial transfers represent another 24% of net national income and are nearly exclusively exchanged within the household (23%). Since neither income nor consumption surveys include information about the providers or recipients of inter-household transfers, they cannot be included in the calculations. Excluding them affects the results in a conservative way. Inter-household transfers are predominantly provided by separated/divorced biological parents or grandparents. If such transfers were included, the parental transfer packages we estimate below would be even larger. Note also that the source of a familial transfer can be a public transfer. For example, unemployed parents use their benefits to support their children. However, such a transaction represents an offsetting effect. The public benefit received diminishes the net transfer burden of the parent, but transferring the resources further to other family members cancels out this decrease.

Our analysis is limited to income flows and does not cover wealth transfers. Lee *et al*. [89] present NTA-based wealth accounts, but their conceptual framework cannot be extended to cover parenthood status as defined here. The cohabitation-based definition of parenthood is sufficient to capture current flows since it excludes only a tiny fragment (about 5%) of private transfers exchanged between households. However, a significant part of wealth transfers changes hands between non-cohabiting people.

### 5.5. Familial time transfers: unpaid household labour

While NTA shines a wider light on intergenerational resource transfers by extending the statistically visible world from public transfers to private money transfers, it stays within the frontiers of the national economy, as reported in SNA. The NTA contribution thus remains incomplete: NTA rearranges SNA but does not consider what could be termed ‘time transfers', the exchange of goods produced and services provided by *unpaid household labour*. Time is the currency of life [50]. It is also key to a more complete grasp of what generations do for each other [26]. There is a growing understanding of the importance of, and the changing patterns in, the time devoted to family duties and other household labour (for a review, see [90]). To shine a yet wider light on resource transfers between generations, NTA needs to be further extended with National Time Transfer Accounts (NTTA).

The estimation is based on time-use surveys. As a first step, the time spent on unpaid production activities is identified, and its age profile is drawn. Second, based on a set of assumptions specified in electronic supplementary material, S1 appendix §S1.4, home production is assigned to its presumed consumers. Third, the value of time spent in unpaid household labour is evaluated. Net time transfers are calculated as the difference between the values of household labour consumed and provided. We use HETUS data and adopt the procedure applied by [91]. Vargha *et al*. [91] published profiles of the value of unpaid household labour and the consumption of its outcome by gender; we required additional details by parenthood status. Unlike the data sources used to construct the age profiles of public and private money transfers, HETUS data are not released as a micro-dataset but as a set of multidimensional tables. These tables offer details about the time spent on an average day and allow us to distinguish between altogether 20 distinct unpaid household labour activities, which were grouped into two summary categories: childcare (including activities that can be performed only for children) and housework (all other activities). The data source allowed crosstabulations of these activities with basic demographic characteristics, such as gender and age, and some limited child-related household information, such as the number of children of age 0 to 6, the number of children of age 7–17, and the exact age of the youngest child. However, no information was available on household size, the age of other household members, or familial relationships.

Another limitation of the dataset is that it does not allow for the estimation of the value of supervisory time [40]. A parent has to stay home with small children who cannot legally be left alone. Time diaries, the main tool of time use surveys, are not designed to directly capture supervision although they can give an indication with the help of the location of the activity. However, the HETUS tables cannot be used for such purposes. Anyway, supervision is not considered as a separate activity in time use surveys. For instance, parents supervise their children even when they sleep at night, yet this is not counted as a secondary work activity. However, when the parents sleep out and have to hire a babysitter for the night, the replacement cost of child supervision can be significant. This limitation of the estimation is partly, but only partly, counterweighted by the extra housework households with young children perform precisely because the parents must spend more time at home while supervising their children. All in all, the non-inclusion of supervisory time distorts our results in a conservative way: if supervisory responsibilities were included, the P/nP ratio would be higher than reported here.

Pricing unpaid household labour is difficult precisely because it is unpaid: there is no market mechanism to evaluate it. Instead, we assigned the selected work activities to the wages of their closest category by the International Standard Classification of Occupations. This method applies the wages of the persons whose job is done (specialist replacement wage approach) instead of the wages of the persons doing the household work (opportunity cost approach). This choice conservatively affects our results since the opportunity cost approach typically assigns a higher value to household labour (particularly tasks done by men) than the replacement wage approach. For the same reason, applying economy-average wages to household labour (generalist replacement wage approach) would also result in a higher P/nP ratio (for details, see electronic supplementary material, S2 appendix §S2.5).

The resulting information set can be employed to construct production age profiles but gives no sufficient ammunition for even the simplest model of the consumption of goods and services provided by household labour. To extend our information base, we imputed the age by gender by household-characteristic information set into the EU-SILC dataset and modelled the intra-household distribution of the outcome of unpaid household labour based on its household rosters. Childcare was assigned to children, and general housework was distributed equally among all household members. In other words, we apply a *per capita* allocation rule for general housework, as there are no intuitively more plausible alternative rules. Take a typical example of unpaid household labour—house cleaning. In theory, we could have tried to distribute the value of time spent cleaning among household members by the size of their respective rooms, the frequency of their use of common rooms, and so on. Needless to say, with current data this would require heroic assumptions. Instead, the *per capita* allocation rule is a simple, intuively plausible solution.

## 6. Baseline empirical analysis

### 6.1. Cross-sectional analysis: overall age profiles and age profiles by parenthood status

**Figure 1.**
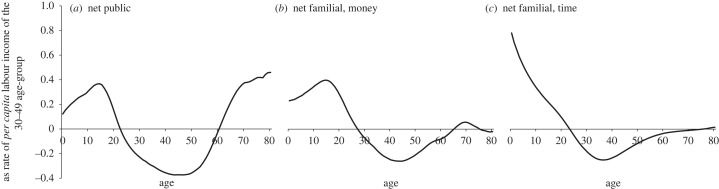
*Per capita* age profiles of net public, net familial money and net time transfers in 12 European countries, 2010. Source: authors' calculations. *Per capita* figures refer to entire year groups, not only those who provide or receive transfers. Values are normalized on the *per capita* market labour income of persons aged 30–49 of the respective country.

Figure 1 shows the age profiles of public, familial money and familial time transfers in net terms (transfers received less transfers provided). They condense information of our 12 European countries. As the aggregation requires re-scaling of the national age profiles, following NTA standards, we use the average market labour income of 30- to 49-year-olds (irrespective of parenthood status and including those who do not work), as presented on the vertical axis of figure 1. The horizontal axes represent ages in cross-section.

The public transfer curve (figure 1*a*) marks three separate age groups. Children and older adults are net beneficiaries; working-age adults are net contributors. This stands in sharp contrast with familial money transfers (figure 1*b*) and time transfers (figure 1*c*). Here, children are net beneficiaries and working-age adults net providers, but the balance for older adults converges to zero. In effect, older age-groups are absent from the intergenerational familial transfer mechanism. Grandparents do not typically live with the families of their adult children in contemporary Europe—and familial transfers are overwhelmingly exchanged within households. Inter-household transfers make up just 5% of the total, both among money and time transfers.

**Figure 2.**
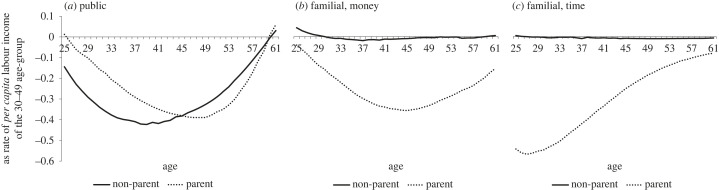
*Per capita* age profiles of net public transfers, net familial money transfers and net familial time transfers by parenthood status in working age in 12 European countries, around 2010. Source: authors' calculation.

Figure 2 replicates figure 1 but is limited to working-age and splits the profiles by parenthood status. The NTA methodology allows a data-driven sectionalization of the life cycle. Since the focus of this study is transfers, we use the net transfer curves to separate age groups (children, working-age people, older people) by their status in transfer provision. Accordingly, people become net transfer providers, all three types of transfers combined, at age 25 and remain in this position until age 61 in the 12-country sample. In other words, ‘resource-dependent childhood' lasts twenty-five years on average in Europe: this is how long it takes for taxpayers and parents to jointly turn a newborn infant into a net ‘resource-productive adult’. Cross-country variation is small: entry age into productive adulthood is 24–25 in all countries except Bulgaria (26) and Spain (27), exit age is 61–62 except in Poland (58), the UK (63) and Sweden (64).

Parents and non-parents do not differ much in terms of their net contributions to public transfers (figure 2*a*). Non-parents pay higher net taxes than parents in more year-groups, and when they do, the gap between the two curves is somewhat larger than when parents take over at age 46. Yet, the overall disparities are not particularly wide. On the whole, non-parents pay more in net taxes than parents do. The real difference comes in familial transfers. Non-parents barely appear to contribute any such transfers either in money or in time, in *net* terms. Non-parents living in childless households do not provide such transfers. Non-parents cohabiting with children do, but they make up less than 3% of the non-parent population. Of course, many childless working-age people also provide valuable upward familial support [42], just like many childless elderly people provide valuable downward contributions. But on aggregate, and as far as data availability allows, *net* familial transfers are provided overwhelmingly, almost exclusively, by parents. Parental money transfers are roughly similar in size to public transfers; parental time transfers are even larger. Consequently, the overall transfer package of parents is significantly larger than that of non-parents. There is a strong class and status gradient to the private resources spent rearing children [27,92,93]. In electronic supplementary material, S4 appendix, we therefore further explored *per capita* age profiles of transfers by education status (below high school, high school graduate, above high school).

### 6.2. Flows-to-stock analysis: the working-lifetime transfer cost of rearing children

Applying the flows-to-stock procedure discussed above, we now use the period age profiles presented in figure 2 as stylized working-lifecourses for parents and non-parents in table 3. The profiles are adjusted with parameter values for economic growth (1.5% annually), mortality (Eurostat *demo_mlifetable_px* table), and a discount rate (5%). We calculate the present values of the expected future net transfers by type (familial time, familial money and public). We express them in terms of the yearly labour income of people between the ages of 30 and 49. Accordingly, the denominator refers to an indicator of the market economy, whereas the numerators include items from both the market economy and the realm of unpaid labour. We call the resulting parent/non-parent ratio (P/nP) the transfer cost of childrearing. In electronic supplementary material, S2 appendix §S2.6, we present the results based on alternative parameter settings.

**Table 3.** Various transfer stocks of parents and non-parents generated over the working life in terms of years of prime-age earnings, 12 European countries in 2010. Source: authors' calculation. *g* = 1.5%; *r* = 5%. Prime-age earnings: average labour income of the 30- to 49-year-old age group. Reported EU12 averages are population-weighted.

	non-parents					parents					parents public/non-parents public	parents total/non-parents total
	(1)	(2)	(3 = 1 + 2)	(4)	(5 = 3 + 4)	(6)	(7)	(8 = 6 + 7)	(9)	(10 = 8 + 9)	(11 = 9/4)	(12 = 10/5)
	familial, time	familial, money	familial, combined	public	total	familial, time	familial, money	familial, combined	public	total		
Belgium	−0.7	−0.2	−0.9	−6.7	−7.6	−8.7	−4.4	−13.1	−5.9	−18.9	0.88	2.49
Bulgaria	−0.2	−0.1	−0.4	−6.9	−7.3	−5.2	−5.9	−11.0	−5.0	−16.1	0.73	2.20
Germany	−0.1	0.1	0.0	−6.6	−6.6	−8.5	−4.2	−12.7	−4.6	−17.3	0.69	2.61
Estonia	0.1	−0.1	0.0	−7.5	−7.5	−5.5	−5.0	−10.5	−5.4	−15.9	0.72	2.10
Spain	−0.3	−0.1	−0.4	−5.3	−5.7	−7.2	−4.8	−12.0	−4.7	−16.7	0.89	2.95
Finland	0.0	0.0	0.0	−6.2	−6.2	−8.9	−5.5	−14.4	−5.4	−19.8	0.87	3.17
France	−0.1	0.0	0.0	−6.0	−6.0	−7.9	−5.0	−12.9	−4.2	−17.1	0.71	2.85
Lithuania	0.0	−0.8	−0.8	−6.5	−7.3	−3.9	−6.3	−10.2	−6.4	−16.6	0.99	2.28
Latvia	0.1	−1.1	−1.0	−7.5	−8.4	−2.7	−5.3	−8.0	−5.4	−13.4	0.73	1.59
Poland	−0.1	−0.3	−0.5	−7.9	−8.4	−9.5	−4.6	−14.1	−5.4	−19.5	0.69	2.32
Sweden	−0.2	−0.1	−0.3	−5.9	−6.2	−8.4	−5.2	−13.6	−4.9	−18.5	0.84	2.99
UK	0.0	0.0	0.0	−6.8	−6.9	−7.3	−5.9	−13.1	−3.5	−16.7	0.52	2.43
coeff. of var. of absolute values	1.16	1.30	0.97	0.11	0.13	0.31	0.12	0.15	0.15	0.10		
EU12	−0.1	0.0	−0.2	−6.4	−6.6	−7.9	−4.9	−12.8	−4.7	−17.5	0.73	2.66

The likely reasons for the cross-country differences in table 3 are multiple. They include, for instance, social policy model traits such as welfare state size, degrees of decommodification and familialism (e.g. [75,77]), levels of economic development, the size and gender composition of the service economy (e.g. [76,94]), wage structure, and the relative importance of the self-subsistence economy. While the number of observations is too small to allow rigorous statistical assessment of the independent effects of various explanatory variables, descriptively, two general messages stand out. They are contained in table 3's summary bottom row showing population-weighted European averages and are replicated in *every* country in the sample.

First, in the public realm, parents everywhere contribute fewer transfers than non-parents over the course of their productive lives: about 4.7 years of prime-age earnings (column 9), compared to 6.4 years for non-parents (column 4). This amounts to about 73% of what non-parents contribute (column 11), ranging from 52% in the UK to sample-highest values of 84% in Sweden, 87–89% in Finland, Belgium and Spain, and 99% in Lithuania. Second, in the family realm, parents, and only parents, everywhere provide in addition a still larger amount of private transfers of money and time. Non-parents barely contribute any familial transfers (0.2 years of prime-age earnings; column 3). But parents contribute total familial transfers that are everywhere significantly larger than their public transfers, typically even two to three times larger: on average, 12.8 years of prime-age earnings (column 8). Descriptively, the total familial contributions by parents range from 8.0 years in Latvia to 12.7–13.1 years in Germany, France, Belgium, and the UK, 13.6 years in Sweden, 14.1 years in Poland, and 14.4 years in Finland. Comparing columns 6 and 7, parental time transfers (7.9 years of prime-age earnings) are on average 1.6 times larger than parental money transfers (4.9 years).

Analytically, table 3 reveals a significant asymmetry in statistical *visibility*. Nearly all non-parental transfers (98%; column 4/column 5) are statistically visible, ranging from 88 to 89% in Belgium, Latvia and Lithuania to 100% in Germany and France. By contrast, only just over one-quarter of parental transfers are statistically visible (column 9/column 10) ranging from 21% in the UK to 39–40% in Lithuania and Latvia. Second, there are significant asymmetries in the sheer *sizes* of the working-lifetime resource transfers to the intergenerational transfer system: those made by parents are about 2.66 times higher than those by non-parents. Descriptively, P/nP ranges from 1.59 in Latvia to 2.61 in Germany, 2.85 in France, 2.95 in Spain, 2.99 in Sweden and 3.17 in Finland. These baseline findings complement studies employing different methods showing that parents have fewer material and time resources than socio-economically comparable non-parents (e.g. [22,24,28,36,37]). But the magnitude of parental contributions, when revealed by including the family realm with NTA and NTTA methods, is much higher here.

Shining a wider light, stepwise, on the relative contributions of parents, beyond merely completing the asymmetries picture, substantially changes it. The P/nP ratio flips around, from 0.73 on average for public transfers alone (column 11) to 1.49 for public and private money transfers combined ((column 7 + column 9)/(column2 + column 4)), to 2.66 for all three transfer types combined (column 12). In line with the asymmetric visibility logic, columns 11 and 12 show that P/nP significantly increases to exceed unity everywhere in our sample. In line with the transfer conversion logic, the coefficient of variance of the absolute values of the overall parental transfer package P in column 10 (0.10) is smaller than that of each of its three subcomponents in columns 6, 7 and 9: time transfers (0.31), money transfers (0.12) and public transfers (0.15). Note also that the same structure of cross-country variance also holds, more strongly, separately for fathers (0.09 versus 0.13, 0.25 and 0.69) and mothers (0.24 versus 0.50, 2.52 and 0.28).

The results reported in table 3 could, in theory, still reflect the economic contributions of unobserved differences between parents and non-parents. But it is robust with regard to the parameters of the flows-to-stock exercise. As electronic supplementary material, S2 appendix §S2.6 shows, lower discount rates and higher economic growth do not affect the patterns described above and change P/nP ratios only marginally. Since we do not directly use the result of the flows-to-stock exercise but compare the outcomes of two such procedures (for parents and non-parents), our conclusions are less vulnerable to the usual risks of estimating stocks from flows. Electronic supplementary material, S5 appendix, therefore further distinguishes between parents with only one and with two or more children. With the exception of Latvia, the difference between single-child parents and multiple-child parents mirrors the baseline difference between non-parents and (all) parents above: single-child parents pay more in public transfers than multiple-child parents, but they contribute less in both private money and private time transfers. Electronic supplementary material, S2 appendix §S2.6, also shows how changes in the cross-sectional age profile would affect the results. Specifically, we quantify the effect of changing parenthood density (equivalent to changing cohort-specific fertility) and find that the results reported here are conservative: in countries where cohort-specific fertilities have changed more, the P/nP ratio is actually smaller.

## 7. Child-rearing as a highly taxed activity?

The baseline findings in table 3 raise the question of whether European societies may implicitly ‘tax' their own reproduction very heavily. Going one step further in the empirical analysis, table 4 therefore estimates the metaphorical tax rates that are implicitly imposed on parents across Europe. It calculates the difference (rather than the ratio) of the same three transfer type stocks of parents minus that of non-parents over the working life, but now relative to the present value of net consumption over the working life. This exercise allows us to calculate net parental transfer contributions in terms of the net amounts spent on consumption, just like value-added taxes and excise taxes (the two typical forms of taxes on consumption in Europe) are calculated. We follow the same steps as made above, using NTA to draw (net-of-taxes) consumption age profiles. To estimate a consumption stock (the present value of consumption over the working life), we use the same procedure and, in the base case, the same parameters as above. We present results generated by alternative parameter settings in electronic supplementary material, S6 appendix. For comparison, column 1 shows that average VAT rates in our 12 country sample, calculated by taking into account the relative weight of commodities charged with different VAT rates, were 12% on average in 2011, ranging from 8 to 9% in Spain, Poland and the UK to 17–18% in Lithuania and France. Column 2 presents the denominator of our parental tax estimation exercise: working-lifetime net consumption. On average, aggregate net consumption over the working life is equivalent to 10.2 years of prime-age labour income in the sample, with relatively lower values in Nordic and Continental countries and somewhat higher values in the UK and southern and east-central European countries except Estonia.

**Table 4.** Estimated implicit taxes on childrearing. Source: authors' calculation. VAT rates: [95]*.* Prime-age earnings: average labour income of the 30- to 49-year-old age group. Reported EU12 averages are population-weighted.

	(1)	(2)	(3)	(4)	(5)	(6)
	average VAT rate, 2011	present value of net consumption over the working life, in years of prime-age labour income	working-lifetime			
			public	familial money	public + familial money	time
			transfers as % of working-lifetime net consumption			
Belgium	11	8.2	−10	51	40	97
Bulgaria	15	12.7	−15	45	30	39
Germany	15	9.4	−22	45	24	90
Estonia	12	9.2	−23	53	31	60
Spain	9	10.9	−5	43	38	64
Finland	10	9.3	−9	59	50	96
France	18	8.7	−20	58	38	89
Lithuania	17	13.5	−1	41	40	29
Latvia	12	11.8	−17	36	18	24
Poland	8	10.3	−24	41	17	91
Sweden	13	7.5	−13	67	55	109
UK	9	12.4	−27	47	21	58
EU12	12	10.2	−17	48	31	77

Columns 3–6 then estimate the implicit tax rates on rearing children, albeit without separating the investment and consumption components of parental inputs. We do so by relating the excess contributions by parents (net of those by non-parents) for the three types of resource transfers (the numerator) to the consumption estimates of column 2 (the denominator). Column 3 reconfirms that the excess parental contributions of public transfers are negative everywhere. Parents pay fewer public transfers than non-parents, on average by an amount equivalent to 17% of working-lifetime consumption, ranging from 1% less in Lithuania to 27% less in the UK. But column 4 adds that parents contribute many more private ‘money' transfers than non-parents (market goods and services), on average about 48% of working-lifetime consumption, ranging from 36 to 41% more in Latvia, Lithuania and Poland to 67% more in Sweden.

These two transfer types are part of the national economy and do not belong to the realm of unpaid household labour. Together, public transfers and familial money transfers by parents above those by non-parents amount to almost one-third of working-life consumption on average. In other words, if the average European parent in our sample, hypothetically, suddenly became a non-parent, they would be able to consume 31% more in goods and services, ranging from 17% in Poland to 50% in Finland and 55% in Sweden. The implicit (metaphorical) tax rate on these two types of childrearing transfers is high by an objective yardstick. Comparing columns 5 and 1 shows that the 31% average tax rate on parental excess transfers of public and familial money resources alone (excluding time transfers) amounts to more than *two-and-a-half times* the average VAT rates in place across Europe in 2011.

We next enter the realm of unpaid household labour. Column 6 in table 4 shows private time transfers as a percentage of net working-lifetime consumption. The excess net contributions by parents in this realm amounts to 77% of working-lifetime consumption on average, ranging from 24 to 29% in Latvia and Lithuania to 96–97% in Finland and Belgium and 109% in Sweden. This can be interpreted metaphorically as a tax on the time spent in rearing children. While it is expressed in terms of consumption in column 6, the currency is different here. In the realm of unpaid household labour, lower transfer burdens translate into alternative forms of time use. The alternative is not more consumption of goods and services produced by unpaid household labour, but rather less production of unpaid household labour and instead more leisure and more paid work. In other words, if the average European parent became, hypothetically, a non-parent, they would suddenly, by sheer virtue of being a non-parent, be able to spend much more time in leisure and in paid work. Comparing columns 6 and 1 shows that the average 77% tax rate on parental time is very large by the yardstick of real-world consumption taxes: it is more than *six times* higher than the average VAT rate applied across Europe.

## 8. A Nordic paradox? 'Family friendly' welfare societies help mothers work, but do not diminish the implicit tax on child-rearing

Table 3 indicates that parents in the two Nordic societies in our sample contribute relatively more, not fewer, overall resources to the intergenerational transfer systems than parents in other European societies. Table 4 further corroborates this finding. The implicit taxes on parental public and money transfers and on time transfers are highest again in Sweden (55% and 109%) and Finland (50% and 96%). This may seem counterintuitive. Nordic Europe's policy models, after all, are famously family friendly, with extensive subsidized childcare facilities with internationally high coverage and low child/carer ratios, and comparatively extensive parental leaves, family allowances, and other social policies to support parents and improve their work–family balance [7,69,75–77]. But as hypothesized in §4.2, the family friendliness of a welfare state does not necessarily imply a reduction of the overall transfer contributions of its parents. Nordic welfare states clearly help parents, especially mothers, through long and generous maternal and parental leave policies to rear their youngest children at home. Thereafter they help them, through generous childcare policies, to go back to paid employment often in relatively well-paid jobs in the large and female-dominated public service sector [74]. This is turn boosts mothers' market incomes and the welfare state's tax base [69,76,77]. Nordic family friendly policies also reduce poverty rates among families with children, gender inequality especially among low earners [74] and long-run father–mother income gaps [29], though they may also reduce mothers' access to high-earning positions and occupations [72,73].

The Nordic welfare states, which also impose high levels of labour income taxes and social security contributions, may not significantly reduce the overall transfer contributions of mothers, but they are likely to make their lives considerably easier. In other words, the help provided by family friendly policy models such as in Nordic Europe is real—but it is not a gift. The general logic of transfer conversion applies here too (§4.2). Nordic welfare states defamilialize mothers and help them fulfil their joint family and career ambitions [75,76]. In so doing, they give a larger degree of freedom to women. But rather than reducing Nordic mothers' resource transfers as child-rearers, these welfare states offer a more extensive alternative public transfer channel.

This also points to the wider role of service sector wages in the estimation of parental and non-parental transfer contributions. Baumol's cost disease [96] (the disproportionate rise in relative service prices as service sector productivity tends to lag behind manufacturing) may not characterize all services. But it is likely to apply to the inherently labour-intensive care services most relevant to parents [11,75,97]. Comparatively high caring sector wages play a double role here. Higher gross caring sector wages are, first, a direct tax burden, for instance in the form of daycarers', kindergarten teachers' and school teachers' wages. But second, they are also used in our valuation method as replacement wages for pricing at-home parental time. To the extent that it is mainly mothers who benefit from caring sector employment, high caring sector wages are thus likely to simultaneously increase the familial time contributions of at-home mothers (thereby increasing the mother/non-mother gap in unpaid household labour) and to increase the public transfer contributions of employed mothers (thereby decreasing the mother/non-mother gap in public transfers).

This means that comparatively more service-intensive welfare states, such as in Nordic Europe, are likely to be more expensive in terms of both the direct cost of childcare (carers' wages) and its replacement cost, leading to higher P/nP ratios. The Nordic ‘solidaristic wage bargaining’ model, in which 'alliances of ends against the middle' managed to boost growth and employment while reducing pretax wage differentials, leads to more compressed wage distributions and thus to higher levels of low-end caring sector wages [98,99]. In other words, by valuing unpaid family alternatives to paid work more, we accurately capture the fact that Nordic societies tend to *value* paid care work more. After all, compared to, say, French or German parents, Nordic parents really do need to pay more if they want to replace their own child care with either public or private carers' wages.

## 9. Parenthood—or motherhood?

**Figure 3.**
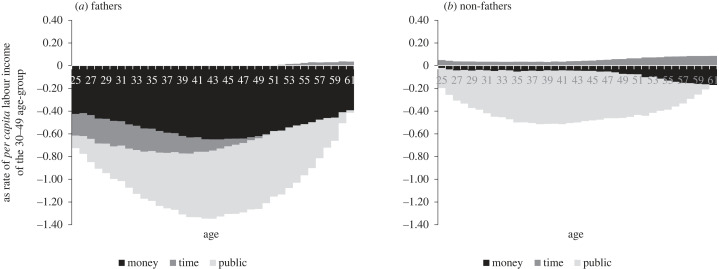
The relative cost of fatherhood: combined transfer packages of men by parenthood status and age. Source: authors' calculation.

**Figure 4.**
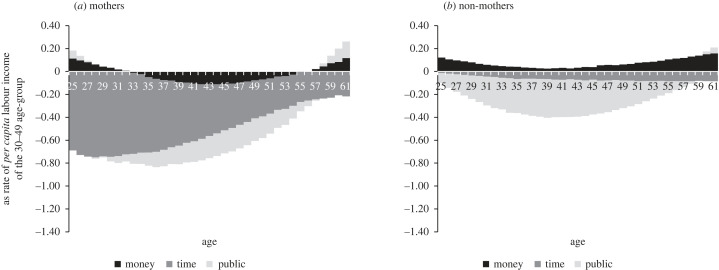
The relative cost of motherhood: combined transfer packages of women by parenthood status and age. Source: authors’ calculation.

Parents, not states (taxpayers), bear the lion's share of the cost of rearing children. Adda *et al*. [22] decompose the life cycle career cost of children into loss of skills during career interruptions, lost earning opportunities, and selection into more child-friendly occupations. But there is an important further gender component to this. As a result of evolved cultural norms and asymmetric power dynamics in gender bargaining at the micro and macro level, all three factors above may affect *mothers* more strongly than fathers [10,12,29,33,35,68,100,101]. It is conceivable that parent/non-parent differences, as analysed above, may in fact prove to be a gender imbalance. To explore these questions, we further split parents into mothers and fathers and non-parents into non-mothers and non-fathers. Figure 3 shows the cross-sectional age profiles of, respectively, public, familial money and familial time transfers in net terms (transfers received less transfers provided) separately for fathers and non-fathers on average for our 12-country sample. Figure 4 does the same for mothers and non-mothers.

Figure 3 shows that fathers contribute somewhat more public transfers, and they do so at higher ages than non-fathers. But the real father/non-father differences appear in transfers in the family realm, which are less observable in public statistics and are largely unrecognized by eligibility rules of public healthcare or pension systems: time transfers and, especially, money transfers. Non-fathers are on average even net beneficiaries of time transfers in all working-age groups, by an amount that almost mirrors their net donation of money transfers. Hence non-fathers' intra-household familial transfers are practically zero at all working ages. By contrast, fathers pay significant money transfers that support the consumption of both mothers and children. Fathers also contribute net time transfers while their children are small, but they subsequently become minor net beneficiaries after age 52.

Figure 4 shows that the composition of the packages of mothers and non-mothers is different from those of fathers and non-fathers. While fathers contribute more public transfers than non-fathers, mothers contribute fewer public transfers than non-mothers. By contrast, mothers' private transfers are significantly larger. Non-mothers contribute minor time transfers but are net recipients of money transfers, making their net resource contribution almost exclusively public at all working ages. By contrast, mothers' contributions are mostly in the less visible family realm and, within it, overwhelmingly in terms of time. The high overall contributions by parents relative to non-parents in figure 2 and table 3 are shown here not to be just higher contributions by mothers. The cross-gender differences respond more strongly to changes in the assumptions than the difference between parents and non-parents do. For instance, if we applied a single average wage for the economy in the monetary valuation of unpaid labour (instead of the multiple activity-specific wages we have used), the mother/father gap would diminish or even disappear. But for the same reason, the P/nP ratio would grow even larger than reported above. Electronic supplementary material, S2 appendix §S2.5, gives more details about the robustness of our estimates of time transfers. While there are many more women than men who are co-residing parents, the gap between mothers and non-mothers is not larger than the gap between fathers and non-fathers. Applying a similar flows-to-stock procedure as above, we now use the period age profiles presented in figures 3 and 4 as stylized working-lifecourses by parenthood status and gender.

Table 5 presents the population-weighted averages of the 12-country sample of the various transfers for fathers and non-fathers, and mothers and non-mothers. The marginals are weighted averages by gender and parenthood status. A number of key observations stand out. Fathers and mothers both contribute much more than respectively non-fathers and non-mothers. The working-lifetime transfer burden of fathers is larger than that of non-fathers in each transfer category. The father/non-father gap is larger by a small margin in public transfers (one year of prime-age labour income) and by a larger margin in time transfers (3.7 years). But there is a huge father/non-father gap of ten years (11.2–1.2) in familial money transfers. The working lifetime transfers picture is different for mothers and non-mothers. Non-mothers provide more public transfers than mothers by about 2.9 (4.8–1.9) years of prime-age labour income, but this is almost fully compensated (2.6 years) by money transfers, which mothers contribute more than non-mothers. The real difference (10.4 years) between mothers and non-mothers lies in their provision of time transfers.

**Table 5.** Transfer stocks of parents and non-parents generated over the working life in terms of years of prime-age earnings by gender for three types of resource transfers (public, private money, private time), 12 European countries in 2010. Source: authors' calculation.

	parental status		
	parent	non-parent	total
**public**			
women	−1.9	−4.8	−2.9
men	−8.4	−7.4	−8.2
total	−4.8	−6.2	−5.4
**money**			
women	−1.2	1.4	0.2
men	−11.2	−1.2	−6.7
total	−5.0	0.1	−3.1
**time**			
women	−11.6	−1.2	−7.6
men	−2.7	1.0	−0.8
total	−7.9	0.0	−4.4

Looking at familial transfers of money and time devoted to unpaid household labour first, a striking feature is how little the aggregates for mothers and fathers differ. What sharply distinguishes fathers and mothers in the family realm is not their overall familial transfer contributions but their composition: fathers mainly contribute market goods and services, mothers mainly contribute unpaid household labour. Sociological work has delved deeper into the contextual causes for such a gendered division of labour (e.g. [70,90,102]). Our analysis further shows why valuing unpaid household labour by extending NTA-based calculations with an NTTA exercise is essential to more fully reveal the extent of mothers' contributions. At the same time, the NTA methodology does justice to parents of both genders. Measuring intrafamilial money transfers makes fathers’ contributions more completely visible; incorporating the realm of unpaid labour does the same with mothers. A similar gender pattern appears among non-parents. The family realms for non-fathers and non-mothers are nearly perfect mirror images. In all age groups, and increasingly so by age, non-fathers provide more familial transfers and non-mothers provide more time transfers. While this might appear to corroborate much-debated specialization-by-gender theories [103], it should be added that gendered roles appear increasingly unrelated to relative earnings potential [30,32].

Overall, gender status informs about the type of invisible transfers provided; parenthood status informs about their magnitude (which in turn depends on how unpaid services are valued relative to paid services). When we next also consider public transfers to eyeball the overall picture of all three types of transfers combined, two observations stand out. First, fathers provide more total transfers than mothers. Second, the gap between fathers and non-fathers is not strikingly different from the gap between mothers and non-mothers. Current methods both of measuring and valuing paid and at-home care work and of distributing consumption among household members may hide inequalities to the detriment of women [12,104]. Yet our findings indicate that gender imbalances may not be, first and foremost, about resource consumption or production. Instead, gender imbalances reflect deeper asymmetries in property rights, eligibilities, and valuation of different types of societally valuable contributions, and therefore, ultimately, in societal norms and power relations.

For a host of sociological and political economy reasons that provide stringent macro-institutional and macro-structural constraints on individual-level preferences, women still record generally lower labour market participation rates, total working hours, and hourly wages than men (e.g. [32,33]). Gender inequalities in all three of these components of earning inequalities tend to increase *after* parenthood, which suggests that unpaid care work plays a key role [30]. For instance, the long-run cost of *mother*hood in earnings alone, while varying, is high everywhere: 21–26% in Scandinavian countries, 31–44% in Anglo-Saxon countries and 51–61% in German-speaking countries. By contrast, the long-run cost of *father*hood is zero in Denmark, minor in Sweden and Anglo-Saxon countries, and even negative in German-speaking countries [29]. The third component, lower wages, in part results from women's higher concentration in lower-paying occupations, often in caring work (e.g. [12,94,97,105]). Care sector penalties and motherhood penalties, while ever-evolving and public policy supply-dependent, remain deeply entrenched [29,31,32,34,101,106]. The work of carers, mostly mothers, in reproducing society over time is both societally undervalued and imperfectly accounted for [10,12,25,39,44,45,68].

Better accounting, by more comprehensively valuing all types of productive work, matters crucially not just for equity reasons but also for the efficient allocation of human capabilities to different types of work. Shining a wider light on what working-age adults contribute also inside the family indicates that the source of father–mother inequity is not primarily in how much genders contribute but may instead reflect how their respective contributions are valued. What fathers contribute is largely measured, societally valued, and protected by contracts and property rights; what mothers contribute, especially at home, in rearing the next generation largely is not [10,12]. This tilts intra-household power relations, even when anti-discrimination laws are in place and the legal standing of genders is equal, including the right to inherit or receive education [32,100,105]. As an accounting framework, NTA is not prepared to capture the deeper nature of these gendered power imbalances. As noted, our replacement wage method used the comparatively low market valuation of paid care work. Since it therefore attaches a comparatively low value to at-home care work, it mainly undervalues the contributions, specifically, of mothers.

## 10. Conclusion and implications for policy and society: debunking the ‘stork theory' of child-rearing

This study has measured the intergenerational transfer contributions by people co-residing with their children relative to people either having no children or not co-residing with them, by going beyond net public transfers to also factor in two types of statistically less visible transfers in the family realm: of market goods (money) and unpaid household labour (time). Because of the irreplaceable primary role of parents in rearing minor children in a Europe of two-generational households, the resource packages parents contribute to the intergenerational transfer system are quite radically different from those of non-parents everywhere. Non-parents contribute almost exclusively to public transfers, more so than parents. But parents, and only parents, provide in addition still larger, albeit less visible, familial transfers: mothers mainly unpaid household labour, fathers mainly market goods.

Valuing not just public transfers but also these two types of private transfers has revealed significant asymmetries in statistical visibility. Nearly all non-parental transfers are statistically visible, compared to just over one-quarter of parental transfers. Shining a wider light, stepwise, on the relative contributions of parents does not merely complete the asymmetries picture; it substantially changes it. The average ratio of parental contributions to non-parental contributions flips around: from 0.73 for public transfers only, to 1.49 for public and private money transfers, to 2.66 when we further add time transfers. The invisibility of these private parental transfers indicates that welfare states implicitly freeride on the cost of producing their own future taxbase—the next generation of taxpayers. As we showed, it takes on average 25 years to rear a newborn infant into a net-resource productive adult in Europe. Yet to the degree that current policies and accounting procedures do not take into account how the fiscal resources the welfare state taps into were created in the first place, they implicitly adhere to an erroneous ‘stork theory’ of how productive adults come about. Certainly, this observation does not imply any argument against welfare states as effective channels for intergenerational resource transfers [5]. But it does call into question current practices of redistribution. As long as welfare states, even famously family friendly ones, ignore the full cost of rearing infants into productive adulthood, they invisibly redistribute from parents to non-parents.

The 2.66 value also captures the sheer *magnitude* of the invisible transfer from parents to non-parents. And the imputed ‘tax rates' implicitly imposed thereby exclusively on childrearing are between two-and-a-half and six times higher than the value-added tax rates *de facto* in place across Europe on consumer goods such as food, clothes, electronics and furniture. Asymmetric resource contributions of these magnitudes are not currently part of public policy debates. Whether and to what degree one believes that public policies should compensate parents, it is important to better measure the distributional impact of the status quo as this allows policy debates to be held on more complete terms. Our main aim has been to empirically lay bare the magnitudes of, and the asymmetries in, the contributions of parents relative to non-parents. Revealing their sheer size by more completely measuring the full transfer package is a prerequisite for more accurately assessing whether societies deem the status quo to be desirable or not.

Our findings thus raise important questions about the optimality of current distributions of the cost of societal reproduction between parents and non-parents. Unless an opposite asymmetry in net benefits appears in old age, the scale of these working-life asymmetries constitutes a large *de facto* redistribution from parents to non-parents that is statistically largely hidden from view. To repeat, children are undoubtedly also consumption 'private goods' conferring utility or well-being benefits to their own parents. The policy question regarding parental redistribution/compensation arises from the fact that children are, additionally and significantly, *also* investment 'public goods' to their parents' generation, whose members (including non-parents) will tomorrow depend on today's children's productive contributions.

Ours is a descriptive accounting analysis of the relative working-life cycle patterns of three types of resource transfers contributed by parents and non-parents in Europe around 2010. This analysis has limitations. For instance, we could not estimate the contributions of parental *offspring* over time, though this has led us to err almost by definition on the conservative side in estimating the parents/non-parents gap. Wolf *et al*. [41], who do estimate for parental descendants, find that the ratio of the combined net present value of public taxes paid minus public benefits received by US parents and their offspring exceeds that of non-parents (who have no offspring).

Second, we have not considered parents' indirect role in children's potential future negative impact on the environment, notably through net carbon emissions. As we have noted, children embody also a third component: they are environmental ‘public bads’ to both their own and their parents' generations. In recent years, several authors have argued that parents are not just indirectly causally responsible but also directly morally responsible for their offspring's carbon emissions. Therefore, they claim, a desirable approach to reduce carbon emissions is to penalize parents in some form for procreating [58–60]. Our generational resource-contribution accounting method cannot measure these environmental externalities but does help to reveal an important neglected element in these debates. Since children are indivisible, the positive and negative externalities they produce are inseparable. In policy terms, this means that penalizing parents for the environmental burden of their children, if this were deemed to be societally desirable, would seem to logically imply rewarding parents for the productive contributions of their children (see electronic supplementary material, S3 appendix).

Here is where our approach points to a last implication for policy and society. This study has laid bare the sheer scale of the asymmetric transfer *contributions* by parents. Let us note, in ending, a further asymmetry in the way societies *reward* alternative forms of saving for late life [10,47,53]. The returns to one form (private savings in personal financial assets) are privatized; those to another form (parental investments in the productive capabilities of children) are largely socialized. After all, the scarce resources which parents spent on children could have been put to alternative uses, such as increased leisure or consumption or positive-return investments such as assets or savings accounts. Technically, older persons' public transfers could be conceived, in part or fully, as returns to their earlier investment in rearing children [49,55,107–109]. But empirically, public policies (say, public pensions, health or long-term care benefits) significantly taking into account such past parental investments cannot be observed in contemporary societies. Everywhere, the societal returns of parental childrearing are, by and large, shared with non-parents, concomitantly reducing the benefits available to parents. Larger contributions *and* smaller rewards: this appears to add up to a double whammy on the plates of those who rear children. Although it may be largely hidden from view, the full cost of reproducing society in contemporary Europe seems unequally distributed and rather high.

## Data Availability

We have not collected data but used the European microdata infrastructure. Specifically, we used the European Union Statistics on Income and Living Conditions (EU-SILC), the harmonized European Household Budget Surveys (HBS), the Harmonized European Time Use Survey (HETUS), and for health-related data, the European Health Interview Survey (EHIS). These comparative datasets are collected by the national statistical agencies of the Member States of the European Union (EU) under the supervision of the EU's statistical agency, Eurostat. The protocols of the surveys in question go through ethical checks concerning information and consent of respondents as well as anonymization of released data. Macrodata used in this paper can be downloaded from the site of the statistical agency of the European Union, Eurostat. Variable names are indicated in the paper. Microdata are publicly available upon request from Eurostat (contact via ESTATMicrodata-access@ec.europa.eu) but cannot be transferred to a third party. To apply for access to Eurostat's microdata, a researcher's organization must first be recognized as a research entity, a university, research institution, or research department in a public administration, bank, statistical institute etc. Applications for research entity recognition should be sent to ESTATENTITIESASSESSMENT@ec.europa.eu. A full replication package is available at Dryad Digital Repository: https://doi.org/10.5061/dryad.wstqjq2s5 [110]. The data are provided in electronic supplementary material [111].

## References

[1] Ando A, Modigliani F. 1963 The ‘Life Cycle’ hypothesis of saving: aggregate implications and tests. Am. Econ. Rev. **53**, 55-84.

[2] Becker GS, Murphy KM. 1988 The family and the state. J. Law Econ. **31**, 1-18. (10.1086/467147)

[3] Samuelson PA. 1958 An exact consumption-loan model of interest with or without the social contrivance of money. J. Polit. Econ. **66**, 467-482. (10.1086/258100)

[4] Lee R, Mason A. 2011 Population aging and the generational economy. Cheltenham UK: Edward Elgar.

[5] Vanhuysse P, Medgyesi M, Gál RI. 2021 Welfare states as lifecycle redistribution machines. PLoS ONE **16**, e0255760. (10.1371/journal.pone.0255760) 34432792 PMC8386825

[6] Vanhuysse P, Gál RI. 2023 Intergenerational resource transfers in the context of welfare states. In The Oxford handbook of family policy: a life-course perspective (eds M Daly, B Pfau-Effinger, N Gilbert, D Besharov), pp. 1015-1033. Oxford, UK: Oxford University Press.

[7] Kolk M. 2021 Government transfers to parents and population policy in a global perspective: an economic demographic approach. J. Dev. Stud. **57**, 1483-1498.

[8] Kneeland H. 1929 Woman's economic contribution in the home. Ann. Am. Acad. Polit. Soc. Sci. **143**, 33-40. (10.1177/000271622914300105)

[9] Reid M. 1934 Economics of household production. New York, NY: Wiley.

[10] Burggraf S. 1997 The feminine economy and economic man. Reading, MA: Addison-Wesley.

[11] Folbre N. 2008 When a commodity is not exactly a commodity. Science **319**, 1769-1770. (10.1126/science.1153904) 18369128

[12] Folbre N. 2020 The rise and decline of patriarchal systems. London, UK: Verso.

[13] Fraser N. 2022 Cannibal capitalism. London, UK: Verso Books.

[14] Perez CC. 2019 Invisible women: exposing data bias in a world designed for men. London, UK: Vintage Books.

[15] Daniels AK. 1987 Invisible work. Soc. Prob. **34**, 403-415. (10.2307/800538)

[16] Bowles S. 2016 The moral economy. New Haven, CT: Yale University Press.

[17] Howard C. 1997 The hidden welfare state. Princeton, NJ: Princeton University Press.

[18] Sen A, Fitoussi JP, Stiglitz J. 2010 Mismeasuring our lives: why GDP doesn't add up. New York, NY: The New Press.

[19] Dasgupta P. 2019 Time and the generations. New York, NY: Columbia University Press.

[20] Putnam R. 2015 Our kids. New York, NY: Simon and Shuster.

[21] Bezzo FB, Raitano M, Vanhuysse P. 2023 Beyond human capital: how does parents' direct influence on their sons’ earnings vary across 8 OECD countries? Oxford Econ. Pap. **75**, gpad007. (10.1093/oep/gpad007)

[22] Adda J, Dustmann C, Stevens K. 2017 The career costs of children. J. Polit. Econ. **125**, 293-337. (10.1086/690952)

[23] Bradbury B. 2008 The time and the cost of children. Rev. Income Wealth **54**, 305-323. (10.1111/j.1475-4991.2008.00277.x)

[24] Doepke M, Zilibotti F. 2019 Love, money, and parenting. Princeton, NJ: Princeton University Press.

[25] Folbre N. 2001 The invisible heart. New York, NY: The New Press.

[26] Gál RI, Vanhuysse P, Vargha L. 2018 Pro-elderly welfare states within child-oriented societies. J. Eur. Public Policy **25**, 944-958. (10.1080/13501763.2017.1401112)

[27] Kornrich S, Furstenberg F. 2013 Investing in children: changes in parental spending on children, 1972–2007. Demography **50**, 1-23. (10.1007/s13524-012-0146-4) 22987208

[28] Penne T, Hufkens T, Goedeme T, Storms B. 2020 To what extent do welfare states compensate for the cost of children? J. Eur. Soc. Policy **30**, 79-94. (10.1177/0958928719868458)

[29] Kleven H, Landais C, Posch J, Steinhauer A, Zweimüller J. 2019 Child penalities across countries: evidence and explanations. Am. Econ. Rev. **109**, 122-126.

[30] Andrew A, Bandiera O, Costa-Dias M, Landais C. 2021 Women and men at work. IFS deaton review of inequalities. London, UK: Institute for Fiscal Studies. See https://ifs.org.uk/inequality/women-and-men-at-work.

[31] Doren C. 2019 Which mothers pay a higher price? Education differences in motherhood wage penalties by parity and fertility timing. Sociol. Sci. **6**, 684-709. (10.15195/v6.a26) 32337322 PMC7182345

[32] Goldin C. 2021 Career and family: women's century-long journey toward equity. Princeton, NJ: Princeton University Press.

[33] Goldin C, Pekkala Kerr S, Olivetti C. 2022 When the kids grow up: women's employment and earnings across the family cycle. NBER Working Paper, report no. 30323.

[34] Kahn JR, García-Manglano J, Bianchi SM. 2014 The motherhood penalty at midlife: long-term effects of children on women's careers. J. Marriage Family **76**, 56-72. (10.1111/jomf.12086) PMC4041155 24904185

[35] Kleven H, Landais C, Leite-Mariante G. 2023 The child penalty atlas. NBER Working Paper, report no. 31649.

[36] Verbist G, Van Lancker W. 2016 Horizontal and vertical equity objectives of child benefit systens: an empirical assessment for European Countries. Soc. Indicators Res. **128**, 1299-1318. (10.1007/s11205-015-1080-9)

[37] Goodin R, Rice J, Parpo A, Eriksson L. 2008 Discretionary time. Cambridge, UK: Cambridge University Press.

[38] Lee R, Miller T. 1990 Population policy and externalities to childbearing. Ann. Am. Acad. Polit. Soc. Sci. **510**, 17-32. (10.1177/0002716290510001002)

[39] Folbre N. 2008 Valuing children. Cambridge, MA: Harvard University Press.

[40] Suh J, Folbre N. 2016 Valuing unpaid child care in the U.S.: a prototype satellite account using the American time use survey. Rev. Income Wealth **62**, 668-684. (10.1111/roiw.12193)

[41] Wolf DA, Lee RD, Miller T, Donehower G, Genest A. 2011 Fiscal externalities of becoming a parent. Popul. Dev. Rev. **37**, 241-266. (10.1111/j.1728-4457.2011.00410.x) 21760651 PMC3134288

[42] Pessando LM. 2019 Childlessness and upward intergenerational support: cross-national evidence from 11 European countries. Ageing Soc. **39**, 1219-1254. (10.1017/S0144686X17001519) 31130759 PMC6532053

[43] Sobotka T. 2017 Childlessness in Europe. In Childlessness in Europe: contexts, causes, and consequences (eds M Kreyenfeld, D Konietzka), pp. 17-55. Cham, Switzerland: Springer.

[44] England P, Folbre N. 1999 The cost of caring. Ann. Am. Acad. Polit. Soc. Sci. **561**, 39-51. (10.1177/000271629956100103)

[45] England P, Folbre N. 1999 Who should pay for the kids? Ann. Am. Acad. Polit. Soc. Sci. **653**, 194-207. (10.1177/000271629956300112)

[46] Folbre N. 1994 Children as public goods. Am. Econ. Rev. **84**, 86-90.

[47] George R. 1987 Who should bear the cost of children? Public Aff. Q. **1**, 1-42.

[48] Alstott AL. 2004 No exit. Oxford, UK: Oxford University Press.

[49] Olsaretti S. 2013 Children as public goods. Phil. Public Aff. **41**, 227-258. (10.1111/papa.12019)

[50] Krueger A, Kahneman D, Schkade D, Schwarz N, Stone A. 2009 National time accounting: the currency of life. In Measuring the subjective wellbeing of nations (ed. A Krueger), pp. 9-86. Chicago, IL: University of Chicago Press.

[51] Gershuny J. 2000 Changing times: work and leisure in postindustrial society. Oxford, UK: Oxford University Press.

[52] Guryan J, Hurst E, Kearney M. 2008 Parental education and parental time with children. J. Econ. Perspect. **22**, 23-46. (10.1257/jep.22.3.23)

[53] Olsaretti S. 2022 Egalitarian justice, population size, and parents' responsibility for the costs of children. In The Oxford handbook of population ethics (eds G Arrhenius, K Bykvist, T Campbell, E Finneron-Burns), pp. 407-429. New York, NY: Oxford University Press.

[54] Deaton A, Stone A. 2014 Evaluative and hedonic wellbeing among those with and without children at home. Proc. Natl Acad. Sci. USA **111**, 1328-1333. (10.1073/pnas.1311600111) 24474755 PMC3910586

[55] Demeny P. 1987 Re-linking fertility behavior and economic security in old age. Popul. Dev. Rev. **13**, 128-132. (10.2307/1972124)

[56] Casal P, Williams A. 1995 Rights, equality and procreation. Analyse Kritik. **17**, 93-116. (10.1515/auk-1995-0107)

[57] Casal P, Williams A. 2004 Equality of resources and procreative justice. In Dworkin and his critics (ed. J Burley), pp. 150-169. Malden, MA: Blackwell.

[58] MacIver C. 2015 Procreation as appropriation. In Permissible progeny? The morality of procreation and parenting (eds S Hannan, S Brennan, R Vernon), pp. 107-129. Oxford, UK: Oxford University Press.

[59] Hedberg T. 2018 Climate change, moral integrity, and obligations to reduce individual greenhouse gas emissions. Ethics Policy Environ. **21**, 64-80. (10.1080/21550085.2018.1448039)

[60] Conly S. 2016 One child. Do we have a right to more? Oxford, UK: Oxford University Press.

[61] Cohen J, Eiduson BT. 1976 Changing patterns of childrearing in alternative life-styles. In Child personality and psychopathology, vol. 3 (ed. A Davids), pp. 25-68. New York, NY: Wiley.

[62] Abramitzky R. 2018 The mystery of the kibbutz. Princeton, NJ: Princeton University Press.

[63] Wade M, Fox N, Zeanah CH. 2019 Long-term effects of institutional rearing, foster care, and brain activity on memory and executive functioning. Proc. Natl Acad. Sci. USA **116**, 1808-1813. (10.1073/pnas.1809145116) 30642973 PMC6358707

[64] Nelson C, Fox N, Zeanah C. 2014 Romania's abandoned children. Cambridge, MA: Harvard University Press.

[65] Hrdy SB. 1999 Mother nature. New York, NY: Pantheon.

[66] Hrdy SB. 2009 Mothers and others: the evolutionary origins of mutual understanding. Cambridge, MA: Harvard University Press.

[67] Verbist G, Diris R, Vandenbroucke F. 2020 Solidarity between generations in extended families: old-age income as a way out of child poverty? Eur. Sociol. Rev. **36**, 317-332. (10.1093/esr/jcz052)

[68] Orloff AS. 1993 Gender and the social rights of citizenship: the comparative analysis of gender relations and welfare states. Am. Sociol. Rev. **58**, 303-328. (10.2307/2095903)

[69] Gornick JC, Meyers MK. 2003 Families that work: policies for reconciling parenthood and employment. New York, NY: Russell Sage.

[70] Hook J. 2006 Care in context: men's unpaid work in 20 countries, 1965–2003. Am. Sociol. Rev. **71**, 639-660. (10.1177/000312240607100406)

[71] Hook J, Paek E. 2020 National family policies and mothers' employment: how earnings inequality shapes policy effects across and within countries. Am. Sociol. Rev. **85**, 381-416. (10.1177/0003122420922505) 33612841 PMC7891546

[72] Mandel H, Semyonov M. 2005 Family policies, wage structures, and gender gaps: sources of earnings inequality in 20 countries. Am. Sociol. Rev. **70**, 949-967. (10.1177/000312240507000604)

[73] Mandel H, Semyonov M. 2006 A welfare state paradox: state intervention and women's employment opportunities in 22 countries. AJS **111**, 1910-1949. (10.1086/499912)

[74] Mandel H, Shalev M. 2009 How welfare states shape the gender pay gap: a theoretical and comparative analysis. Soc. Forces **87**, 1873-1912. (10.1353/sof.0.0187)

[75] Esping-Andersen G. 1999 Social foundations of postindustrial economies. Oxford, UK: Oxford University Press.

[76] Esping-Andersen G. 2009 The incomplete revolution. Cambridge, UK: Polity Press.

[77] Esping-Andersen G. 2016 Families in the 21st century. Stockholm, Sweden: SNS Förlag.

[78] UNICEF. 2019 Supporting families and providing early childhood education and care in Europe and Central Asia: policy and financing options. Geneva, Switzerland: UNICEF.

[79] 2011 European Union Statistics on Income and Living Conditions [Internet]. See https://ec.europa.eu/eurostat/web/microdata/european-union-statistics-on-income-and-living-conditions.

[80] 2011 Household Budget Survey scientific-use files [Internet]. See https://ec.europa.eu/eurostat/web/microdata/household-budget-survey.

[81] 2000 Harmonized European Time Use Surveys [Internet]. See https://ec.europa.eu/eurostat/web/microdata/harmonised-european-time-use-surveys.

[82] 2022 European Health Interview Survey [Internet]. See https://ec.europa.eu/eurostat/web/microdata/european-health-interview-survey.

[83] Bommier A, Lee R. 2003 Overlapping generations model with realistic demography. J. Popul. Econ. **16**, 135-160. (10.1007/s001480100102)

[84] Lee R. 1994 The formal demography of aging, transfers, and the economic life cycle. In The demography of aging (eds LG Martin, H Preston Samuel), pp. 8-49. Washington, DC: National Academy Press.

[85] Lee R. 1994 Population age structure, intergenerational transfers, and wealth. J. Hum. Res. **29**, 1027-1063. (10.2307/146133)

[86] Lee RD, Mason A. 2011 Theoretical aspects of National Transfer Accounts. In Population ageing and the generational economy (eds RD Lee, A Mason). Cheltenham, UK: Edward Elgar.

[87] United Nations. 2013 National transfer accounts manual. New York, NY: United Nations.

[88] Istenič T, Hammer B, Šeme A, Lotrič-Dolinar A, Sambt J. 2017 European national transfer accounts manual. Vienna, Austria: Wittgenstein Centre.

[89] Lee R, McCarthy D, Sefton J, Sambt J. 2017 Full generational accounts: what do we give to the next generation? Popul. Dev. Rev. **43**, 695-720. (10.1111/padr.12113)

[90] Cornwell B, Gershuny J, Sullivan O. 2019 The social structure of time: emerging trends and new directions. Annu. Rev. Sociol. **45**, 301-320. (10.1146/annurev-soc-073018-022416)

[91] Vargha L, Gál RI, Crosby-Nagy MO. 2017 Household production and consumption over the life cycle: national time transfer accounts in 14 European countries. Demogr. Res. **36**, 905-944. (10.4054/DemRes.2017.36.32)

[92] Jackson MI, Schneider D. 2022 Public investments and class gaps in parents' developmental expenditures. Am. Sociol. Rev. **87**, 105-142. (10.1177/00031224211069975) 36860991 PMC9974177

[93] Schneider D, Hastings OP, LaBriola J. 2018 Income inequality and class divides in parental investments. Am. Sociol. Rev. **83**, 475-507. (10.1177/0003122418772034)

[94] England P. 2010 The gender revolution: uneven and stalled. Gender Soc. **24**, 149-166. (10.1177/0891243210361475)

[95] CPB. 2013 A study on the economic effects of the current VAT rates structure. Addendum: country analyses. The Hague, The Netherlands: CPB Netherlands Bureau for Economic Policy Analysis.

[96] Baumol W. 1967 The macroeconomics of unbalanced growth. Am. Econ. Rev. **57**, 415-426.

[97] England P, Budig M, Folbre N. 2002 Wages of virtue: the relative pay of care work. Soc. Probl. **49**, 455-473. (10.1525/sp.2002.49.4.455)

[98] Moene KO, Wallerstein M. 1997 Pay inequality. J. Labor Econ. **15**, 403-430. (10.1086/209866)

[99] Moene KO, Wallerstein M. 2003 Earnings inequality and welfare spending: a disaggregated analysis. World Polit. **55**, 485-516. (10.1353/wp.2003.0022)

[100] Folbre N. 2006 Chicks, hawks, and patriarchal institutions. In Handbook of contemporary behavioral economics (ed. M Altman). Armonk, NY: M.E. Sharpe.

[101] Glauber R. 2018 Trends in the motherhood wage penalty and fatherhood wage premium for low, middle and high earners. Demography **55**, 1663-1680. (10.1007/s13524-018-0712-5) 30255427

[102] Killewald A. 2013 A reconsideration of the fatherhood premium: marriage, co-residence, biology, and fathers’ wages. Am. Sociol. Rev. **78**, 96-116. (10.1177/0003122412469204)

[103] Becker GS. 1981 A treatise on the family. Cambridge, MA: Harvard University Press.

[104] World Bank. 2018 Poverty and shared prosperity 2018. Washington, DC: World Bank.

[105] Iversen T, McCall Rosenbluth F. 2010 Women, work, and politics: the political economy of gender inequality. New Haven, CT: Yale University Press.

[106] Budig M, England P. 2001 The wage penalty for motherhood. Am. Sociol. Rev. **66**, 204-225. (10.1177/000312240106600203)

[107] Sinn HW. 2004 The pay-as-you-go pension system as fertility insurance and an enforcement device. J. Public Econ. **88**, 1335-1357. (10.1016/S0047-2727(03)00015-X)

[108] Cigno A, Werding M. 2007 Children and pensions. Cambridge, MA: MIT Press.

[109] Coleman JS. 1993 The rational reconstruction of society: 1992 presidential address. Am. Sociol. Rev. **58**, 1-15. (10.2307/2096213)

[110] Vanhuysse P, Medgyesi M, Gal RI. 2023 Data from: Taxing reproduction: the full transfer cost of rearing children in Europe. Dryad Digital Repository. (10.5061/dryad.wstqjq2s5) PMC10565363 37830014

[111] Vanhuysse P, Medgyesi M, Gal RI. 2023 Taxing reproduction: the full transfer cost of rearing children in Europe. Figshare. (10.6084/m9.figshare.c.6858166) PMC10565363 37830014

